# Catalytic and functional aspects of different isozymes of glycolate oxidase in rice

**DOI:** 10.1186/s12870-017-1084-5

**Published:** 2017-08-08

**Authors:** Zhisheng Zhang, Xiangyang Li, Lili Cui, Shuan Meng, Nenghui Ye, Xinxiang Peng

**Affiliations:** 1 Southern Regional Collaborative Innovation Center for Grain and Oil Crops in China, Hunan Agricultural University, Changsha, Hunan 410128 China; 2 State Key Laboratory for Conservation and Utilization of Subtropical Agro-bioresources, College of Life Sciences, South China Agricultural University, Guangzhou, Guangdong 510642 China

**Keywords:** Glycolate oxidase isozymes, Enzymatic characteristics, Physiological functions, Rice (*Oryza sativa*)

## Abstract

**Background:**

Glycolate oxidase (GLO) is a key enzyme for photorespiration in plants. There are four GLO genes encoding and forming different isozymes in rice, but their functional differences are not well understood. In this study, enzymatic and physiological characteristics of the GLO isozymes were comparatively analyzed.

**Results:**

When expressed heterologously in yeast, GLO1, GLO4 and GLO1 + 4 showed the highest activities and lowest *K*_*m*_ for glycolate as substrate, whereas GLO3 displayed high activities and affinities for both glycolate and L-lactate, and GLO5 was catalytically inactive with all substrates tested. To further reveal the physiological role of each GLO isozyme in plants, various *GLO* genetically modified rice lines were generated and functionally analyzed. GLO activity was significantly increased both in *GLO1* and *GLO4* overexpression lines. Nevertheless, when either *GLO1* or *GLO4* was knocked out, the activity was suppressed much more significantly in *GLO1* knockout lines than in *GLO4* knockout lines, and both knockout mutants exhibited obvious dwarfism phenotypes. Among *GLO3* and *GLO5* overexpression lines and RNAi lines, only *GLO3* overexpression lines showed significantly increased L-lactate-oxidizing activity but no other noticeable phenotype changes.

**Conclusions:**

These results indicate that rice GLO isozymes have distinct enzymatic characteristics, and they may have different physiological functions in rice.

**Electronic supplementary material:**

The online version of this article (doi:10.1186/s12870-017-1084-5) contains supplementary material, which is available to authorized users.

## Background

Glycolate oxidase (GLO, EC 1.1.3.15) is an important peroxisomal FMN-dependent oxidase involved in photorespiration. Plant photorespiration begins with the oxygenating reaction of ribulose 1, 5-bisphosphate carboxylase-oxygenase (Rubisco) in chloroplasts. This process produces a toxic intermediate metabolite phosphoglycolate (2-PG), which is further converted to glycolate by 2-PG phosphatase (PGP). Glycolate is transferred to peroxisomes and oxidized into glyoxylate by GLO with equimolar amount of hydrogen peroxide (H_2_O_2_) released [1–3]. In addition to its metabolic function in photorespiration, GLO has been reported to play roles in plant photosynthetic regulation and stress resistance. Suppression of GLO leads to glyoxylate accumulation and inhibits photosynthesis, while overexpressing GLO confers improved photosynthesis under high light and high temperature in rice [4, 5]. GLO has been significantly induced in cowpea, tobacco and pea under drought stress [6–8], while in rice and barley, GLO was induced notably by pathogen infection [9–11]. Furthermore, because of the high metabolite flux of photorespiration, about 70% of the total H_2_O_2_ in C_3_ plants comes from the oxidation of glycolate as catalyzed by GLO, and this value could be even higher under some stress conditions such as drought and high temperature [3, 12–14]. Therefore, GLO may also play an important role in plant H_2_O_2_-related pathways.

The sequencing of *Arabidopsis thaliana*, *Nicotiana benthamiana* and rice revealed that GLO are encoded by a gene family in these plant species [15, 16]. Moreover, GLO isozymes have been observed in several plant species such as *Arabidopsis thaliana*, maize, and spinach [16–19]. The expressions of isozymes are usually tissue-specific, which may satisfy metabolic behavior of the cells in which each isozyme is expressed [20, 21]. For example, the *Arabidopsis* 1-Amino-cyclopropane-1-carboxylate synthase (ACS) isozymes are biochemically distinct, have tissue-specific expression, and function in different cellular environments for C_2_H_4_ synthesis [21]. The GLO isozymes have been reported to show tissue-specific expression in maize and *Arabidopsis* (e.g., there are two different GLO isozymes exist in the bundle sheath and mesophyll tissues of maize leaves) [17, 22], while the enzymatic and physiological characteristics of their isozymes have not been comparatively studied. It is not well understood why there are different tissue-specific GLO isozymes in these plant species. In addition, GLO isozymes are related with resistances to various stresses, wherein each GLO isozyme may perform different functions. Rojas et al. (2012) found that each *Arabidopsis* GLO isozyme could play different roles in the H_2_O_2_ signal transduction to induce defense responses during the nonhost resistance of *Arabidopsis thaliana* [16]. *Arabidopsis* GOX1 and GOX2 have been reported to perform different functions in the oxidative stress-related cell death [23]. Accordingly, the potential tissue- or environment-specific expression and enzymatic diversity of rice GLO isozymes would be relevant to the distinct physiological function of each GLO isozyme during various biological processes.

A total of four *GLO* genes have been identified in rice genome (i. e., Os03g0786100, Os04g0623500, Os07g0152900 and Os07g0616500, encoding *OsGLO1*, *OsGLO3*, *OsGLO4* and *OsGLO5*), each of which has a peroxisomal targeting signal, PTS1 [24]. In this study, we comparatively investigated the enzymatic characteristics of each GLO isozyme, and furthermore, different genetically modified rice lines of these *GLO* genes were generated and analyzed for functionality. Our results demonstrate that rice GLO isozymes have distinct enzymatic characteristics, and their physiological functions are nonredundant in rice.

## Results

### 1. Transcriptional expression patterns of *GLO* genes and their responses to stresses

**Fig. 1:**
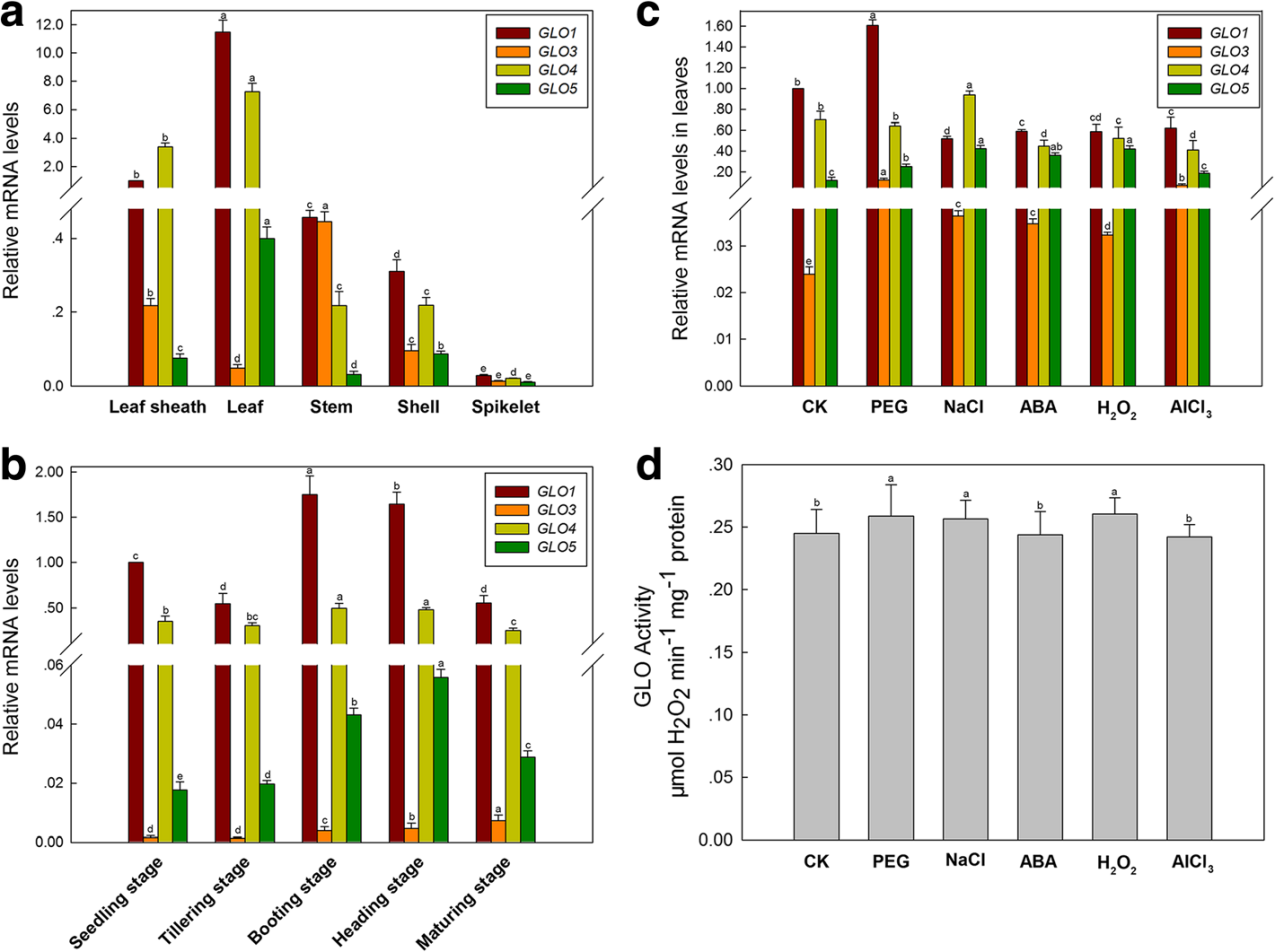
Expression patterns of *GLO* genes and their response to stresses in rice. **a** Relative mRNA levels in different leaf tissues were graphed based on the *GLO1* mRNA level in leaf sheath as 1. **b** Relative mRNA levels at different developmental stages were graphed based on the *GLO1* mRNA level in seedling stage as 1. **c** mRNA transcriptional levels of *GLO* genes in response to various stress treatments for 2 days (PEG 6000, 10%; NaCl, 100 mM; ABA, 10 μM; H_2_O_2_, 5 mM; AlCl_3_, 2 mM; CK, Control group). Relative mRNA levels were graphed based on the *GLO1* mRNA level in CK as 1. **d** GLO activities in response to various stress treatments (the same samples as in Fig.1c). Values are means ± SD (*n* = 3). Means denoted by the same letter did not significantly differ at *P* < 0.05 according to Duncan’s multiple range tests

The rice genome contains four *GLO* genes located on three different chromosomes [15], both the mRNA sequences and polypeptides of these four *GLO* genes are highly similar (Additional file 1). Our previous transcriptional analyses have shown that *GLO1* and *GLO4* were predominantly expressed in leaves, while *GLO3* and *GLO5* were mainly expressed in roots [15, 24]. In this study, we further noticed that *GLO1* and *GLO4* were abundantly expressed in leaves and leaf sheaths, and moderately expressed in stems and husks. *GLO3* was primarily expressed in stems and leaf sheaths, and *GLO5* was only slightly expressed in leaves (Fig. 1a). Furthermore, transcription profiles of *GLO* genes were analyzed for leaves at different growth stages. The expression of *GLO1* increased about 65-75% in the booting and heading stages but not in other developmental periods, while *GLO4* only showed a 35-40% increase in the booting and heading stages (Fig. 1b). The expression levels of *GLO3* and *GLO5* were very low in rice leaves throughout all developmental periods (Fig. 1b).

Because GLO is reported to be involved in stress resistance in plants [6–11], we investigated responses of different *GLO* gene members to various stresses here (PEG 6000, 10%; NaCl, 100 mM; ABA, 10 μM; H_2_O_2_, 5 mM; AlCl_3_, 2 mM; CK, Control group). The results showed that *GLO1* transcripts increased by 60% under PEG treatment, but decreased about 35-40% under other treatments such as NaCl and ABA treatments (Fig. 1c). *GLO4* expression showed a 35% increase under NaCl treatment, but was suppressed by 25-40% under ABA, H_2_O_2_ and AlCl_3_ treatments (Fig. 1c). Elevated expression level of *GLO3* was observed under all treatments, the expression of *GLO5* was also increased under these treatments, except for the AlCl_3_ treatment (Fig. 1c). Meanwhile, GLO activities were correspondingly assayed for the above samples. Inconsistent with the changes in *GLO* gene expression, the GLO activity only increased 5-8% under the PEG, NaCl and H_2_O_2_ treatments (Fig. 1d).

### 2. Enzymatic differences of GLO isozymes

**Fig. 2:**
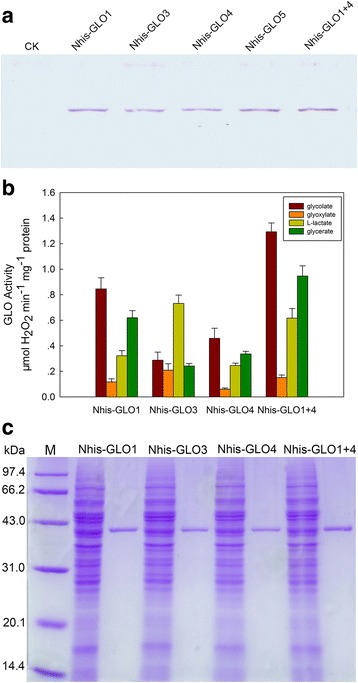
Expression of GLO isoforms in yeast. **a** Western blotting analysis of each GLO isoform expressed in yeast. CK represents the crude enzyme extracted from yeast cells transformed with pYES3/CT vector. Nhis-GLO1, Nhis-GLO3, Nhis-GLO4 and Nhis-GLO5 represent the crude enzyme extracted from yeast cells transformed with pYES3-Nhis-GLO1, pYES3-Nhis-GLO3, pYES3-Nhis-GLO4, pYES3-Nhis-GLO5 respectively. Nhis-GLO1 + 4 represent the crude enzyme extracted from yeast cells co-transformed with pYES3-Nhis-GLO1 and pYES2-Nhis-GLO4. **b** Substrate specificity of each GLO isoform. Values are means ± SD (*n* = 3). **c** The purity and subunit molecular weights of the GLO isoforms purified from yeast. GLO isoforms were purified from yeast cells by immobilized metal affinity chromatography, and the molecular weights of the subunits were determined by uniform SDS-PAGE (12.5%). The results are representative of three independent experiments

Enzymatic characteristics of the GLO isozymes in plants are seldom investigated. Previous research found that there are three types of GLO isozymes present in rice leaves, including two that are homo-oligomers composed of either GLO1 or GLO4 subunits, and the others are hetero-oligomers composed of interacted GLO1 and GLO4 subunits [24]. Here the kinetic properties of rice GLO isozymes were comparatively analyzed. A 6 × His-tag was fused to the N-terminus of each GLO and expressed in the yeast *Saccharomyces cerevisiae*, since it has been proved that the N-terminal His-tag rarely influences the properties of the fused protein [25]. Yeast cell lysates were prepared using acid-washed glass beads, and the western blot analyses showed that all GLO isozymes could be heterologously expressed in *S.cerevisiae* (Fig. 2a). Substrate screens using crude enzyme revealed that GLO1, GLO4 and GLO1 + 4 displayed the highest activity with glycolate as substrate, and showed appreciably high activity on glycerate and less activity on L-lactate and glyoxylate (Fig. 2b). In contrast, GLO3 showed the highest activity on L-lactate, and then on glycolate and glycerate, respectively (Fig. 2b). GLO5 was completely inactive to all substrates tested. Each GLO isozyme was further purified from the yeast crude extraction by immobilized metal-affinity chromatography. SDS-PAGE analysis, which guarantees purity for each isozyme, showed an identical subunit molecular weight of about 40 kDa for various isozymes (Fig. 2c). A preliminary analysis showed that the optimum pH of 7.8 was identical for all GLO isozymes (Additional file 2), and the optimum temperature for GLO1, GLO4 and GLO1 + 4 was 42 °C, while the optimum temperature of GLO3 was 47 °C (Additional file 2).

The kinetic studies were performed at 30 °C and pH 7.8. GLO1, GLO4 and GLO1 + 4 have the highest affinity for glycolate, with *K*_*m(glycolate)*_ values of 0.499 mM, 0.613 mM and 0.423 mM, respectively (Table 1), and the *V*_*max(glycolate)*_ values of GLO1 + 4 and GLO1 were higher than that of GLO4 (Additional file 3). The affinities of GLO1, GLO4 and GLO1 + 4 for glyoxylate, L-lactate and glycerate were much lower than for glycolate (Table 1). GLO3 also exhibited highest affinity to glycolate, meanwhile, it showed a high affinity to L-lactate, with *K*_*m*_ values of 0.470 mM and 1.104 mM, respectively (Table 1). Oxalate is prevalent in the plant kingdom [26], and is a competitive inhibitor of GLO [27]. Here *K*_*i*_ values for oxalate were detected to range from 4.572 to 6.337 mM when using glycolate as substrate (Table 2). Oxalate could more strongly inhibit glyoxylate-oxidizing activity of each GLO isozyme, with *K*_*i*_ values between 1.887 and 3.018 mM (Table 2).

**Table 1:** *K*_*m*_ of each GLO isozyme with various substrates

GLO isozymes	*K*_*m(glycolate)*_ (mM)	*K*_*m(glyoxylate)*_ (mM)	*K*_*m(L-lactate)*_ (mM)	*K*_*m(glycerate)*_ (mM)
GLO1	0.499 ± 0.041^b^	6.505 ± 0.412^a^	5.128 ± 0.315^a^	4.976 ± 0.305^a^
GLO3	0.470 ± 0.035^bc^	1.799 ± 0.084^d^	1.104 ± 0.056^c^	2.762 ± 0.311^c^
GLO4	0.613 ± 0.050^a^	5.983 ± 0.333^b^	4.720 ± 0.291^b^	4.367 ± 0.395^b^
GLO1 + 4	0.423 ± 0.038^c^	4.883 ± 0.321^c^	5.227 ± 0.448^a^	4.511 ± 0.307^b^

Values are means ± SD of three replicates. Means denoted by the same letter did not significantly differ at *P* < 0.05 according to Duncan’s multiple range tests

**Table 2:** *K*_*i*_ of GLO with oxalate

GLO isozymes	*K*_*i(oxalate)*_ (mM)	*K*_*i(oxalate)*_ (mM)
	Glycolate as substrate	Glyoxylate as substrate
GLO1	4.572 ± 0.930^c^	1.887 ± 0.326^c^
GLO3	5.604 ± 1.229^b^	3.018 ± 1.359^a^
GLO4	6.337 ± 1.736^a^	2.272 ± 0.524^b^
GLO1 + 4	5.491 ± 1.041^b^	2.316 ± 0.608^b^

Values are means ± SD of three replicates. Means denoted by the same letter did not significantly differ at *P* < 0.05 according to Duncan’s multiple range tests

### 3. Functional analysis of GLO isozymes

**Fig. 3:**
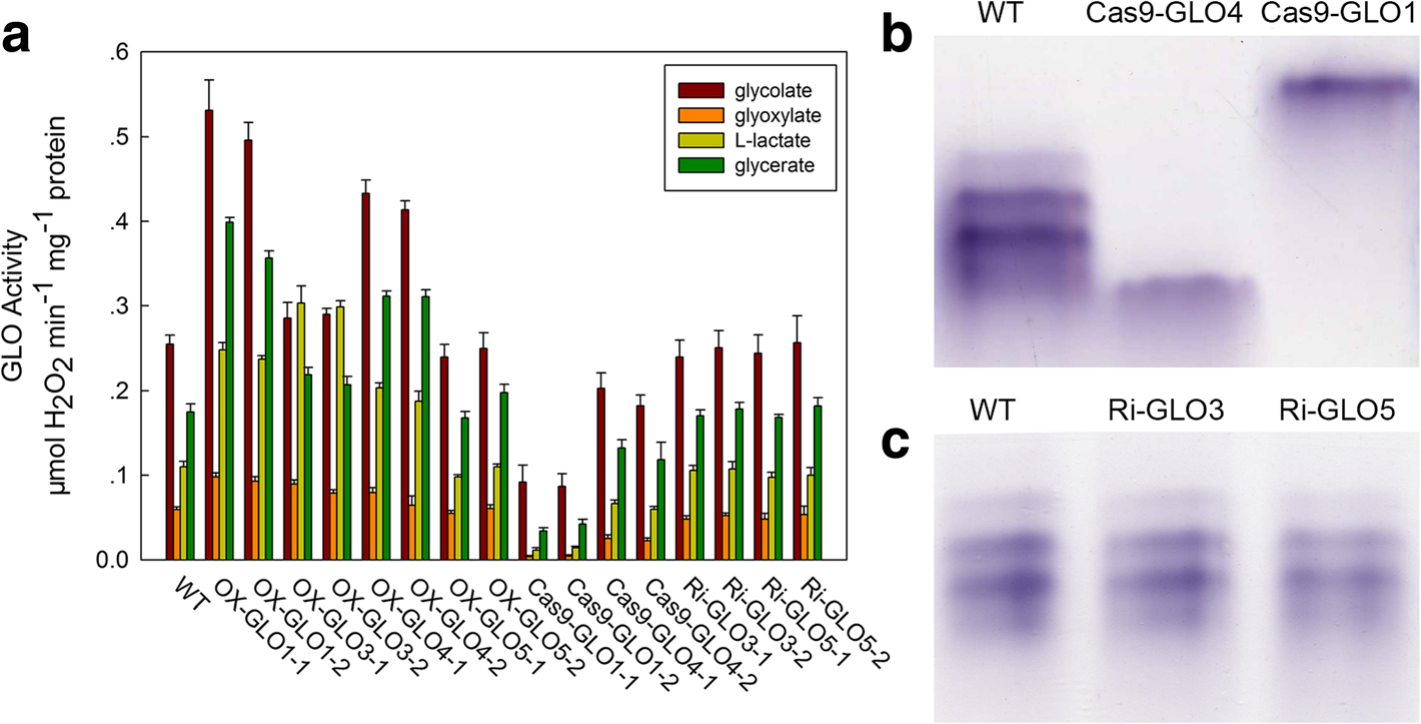
Catalytic characteristics and isozyme patterns of GLO in vivo*.* **a** GLO enzyme activities in different transgenic plants. OX-GLO1, OX-GLO3, OX-GLO4 and OX-GLO5 represent the GLO1, GLO3, GLO4 and GLO5 overexpression transgenic plants, respectively. Cas9-GLO1 and Cas9-GLO4 represent the GLO1 and GLO4 knockout plants, respectively. Ri-GLO3 and Ri-GLO5 represent the specific GLO3 and GLO5 RNA-silencing transgenic plants, respectively. **b** The GLO isozyme bands of WT, Cas9-GLO4 and Cas9-GLO1 plants. **c** The GLO isozyme bands of WT, Ri-GLO3 and Ri-GLO5 plants. The second leaf from the top was detached from plants at 4-leaf stage for determination. Values are means ± SD (*n* = 3)

**Fig. 4:**
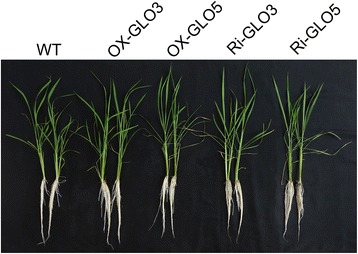
Growth phenotypes of the GLO3 and GLO5 up- or down-regulated transgenic plants. OX-GLO3; OX-GLO5 represent the GLO3 and GLO5 overexpression transgenic plants, respectively; Ri-GLO3 and Ri-GLO5 represent the specific GLO3 and GLO5 RNA-silencing transgenic plants, respectively. The results are representative of three independent experiments

As described above, rice GLO isozymes have distinct enzymatic properties. It is more interesting to know whether these isozymes may play distinct physiological roles in plants. As such, each of the 4 isozymes was overexpressed in rice to determine their contribution to the glycolate metabolism. As shown in Additional file 4, each *GLO* gene was up-regulated as expected at the mRNA level in the corresponding transgenic line. Overexpression of *GLO1* and *GLO4* increased GLO activity by 110% and 65% in rice leaves, respectively. However, overexpression of *GLO3* increased GLO activity by only about 12% (Fig. 3a) but increased the L-lactate-oxidizing activity by more than 140% (Fig. 3a). Overexpression of *GLO5* had no effect on GLO activity, consistent with the result of enzymatic assay. In addition, both *GLO3* and *GLO5* were silenced by RNAi (Additional file 4), *GLO1* and *GLO4* were knocked out using pYLCRISPR/Cas9P_ubi_ system (Additional file 4). In leaves of *GLO1* and *GLO4* knockout lines, GLO activity was decreased by about 65% and 20%, respectively, while suppression of *GLO3* and *GLO5* had no effect (Fig. 3a). The GLO isozymes zymogram analysis of different *GLO* genetically modified rice lines verified that GLO1 and GLO4 were completely knocked out in the corresponding transgenic lines (Fig. 3b), further supporting our previous results [24]. In contrast, suppression of *GLO3* and *GLO5* did not alter GLO isozyme patterns (Fig. 3c), implying that in leaves of wild rice plants (WT), *GLO3* and *GLO5* may have not contributed to GLO activities. Consistent with changes in GLO activities as addressed above, phenotypes were not obviously altered in all the *GLO3* and *GLO5* up-regulated or down-regulated transgenic lines (Fig. 4). However, similar to our previous observations [5], the rice lines with GLO activities suppressed displayed dwarfism phenotype (data not shown), and reduced H_2_O_2_ content was also detected in these rice lines (Additional file 5). However, it was noticed that GLO3 had high activity on L-lactate, meaning that it might participate in the L-lactate metabolism in rice, particularly in the roots (Fig. 2b; Table 1). So we tested if GLO3 contributed to L-lactate tolerance as recently reported by Engqvist et al. (2015). As shown in Additional file 6, the phenotype of *GLO3* overexpression and RNAi lines were not different from that of WT plants under lactate treatment, though lactate-oxidizing activity was markedly increased in both leaves and roots of the overexpression lines (Fig. 3a; Additional file 7).

## Discussion

Members of a gene family can have different tissue-, development- or environment-specific expression patterns. Using the *GLO* gene family that is present in the rice genome [15, 24], we show that the four rice *GLO* genes are differently expressed in various tissues and developmental stages of the rice plant (Fig. 1a and b), as well as in response to stresses (Fig. 1c). Our findings suggest that the physiological roles of these *GLO* genes are not redundant, however, the exact biological significance of the rice *GLO* gene family needs further investigation.

Generally speaking, gene family members that have different expression patterns would encode various isozymes with diverse enzymatic characteristics and physiological roles [21, 28]. In plants, GLO was first purified from spinach [29, 30], the primary structure of spinach GLO (SpGLO) was identified by peptide sequencing, which contains only one uniform peptide [31], and its *K*_*m*_ for glycolate was detected to be 0.38 mM [32]. GLO was also isolated from *Parthenium hysterophorus* and *Pisum sativum*, which both consisted of two different peptides. The *K*_*m(glycolate)*_ of the *Parthenium hysterophorus* GLO is 0.2 mM and that of the *Pisum sativum* GLO is 0.3 mM [33]. However, the enzymatic characteristics of each GLO isozyme in plants are seldom comparatively analyzed. *Arabidopsis* contains five GLO members, i. e. GOX1, GOX2, GOX3, HAOX1 and HAOX2 [4, 34], only GOX1, GOX2 and GOX3 have been heterologously expressed and purified in *E.coli,* respectively. Enzymatic assays of these purified enzymes showed that they all had high affinity for glycolate but with distinct *K*_*m*_ values. In addition, GOX3 possesses high catalytic efficiency for L-lactate [22]. In this study, we comparatively investigated enzymatic characteristics of all rice GLO isozymes. GLO1 + 4 showed highest catalytic efficiency using glycolate as substrate, followed by GLO1 and GLO4, respectively (Fig. 2b; Table 1 and Additional file 3). Indeed, the transgenic rice plants that had up-regulated or knocked-out GLO1 or GLO4 exhibited much higher or lower GLO activity, respectively. Our previous studies also observed that the phenotypes were altered in the GLO1 and GLO4 up-regulated or down-regulated transgenic lines [4, 5]. In contrast, there were no changes in the GLO activity and phenotypes in GLO3 and GLO5 up-regulated or RNAi rice lines (Fig. 3a; Fig 4). In addition, the GLO isozymes zymogram analysis further supported that the GLO isozymes in rice leaves consisted of GLO1 and GLO4 subunits (Fig. 3b and c), and GLO1 was more abundant than GLO4 in rice leaves (Fig. 3b). In combination with our previous results [24], it can be concluded that GLO1, GLO4 and GLO1 + 4 are the GLO isozymes for photorespiration in rice. In addition, this study further revealed that GLO1 + 4 and GLO1 have higher catalytic efficiency on glycolate-oxidation than GLO4 (Fig. 2a and b; Table 1). Therefore it can be further implied that GLO1 could be the major contributor to GLO activity and consequently to photorespiration and the associated H_2_O_2_ production.

GLO3 is the rice homolog of *Arabidopsis* GOX3. It was recently reported that GOX3 in *Arabidopsis* functions as an L-lactate oxidase catalyzing the conversion of L-lactate to pyruvate, in order to maintain low levels of L-lactate in roots under normoxic conditions [22]. We observed that the rice GLO3 was also able to efficiently catalyze the oxidation of L-lactate to pyruvate (Fig. 2b; Table 1). Our previous results have demonstrated that *GLO3* is predominantly expressed in roots [15, 24], but unexpectedly no GLO activities could be detected in wild type rice roots (Additional file 7). We further noticed that the GLO3 overexpression rice plants conferred no improved resistance to L-lactate toxicity (Additional file 6). This was inconsistent with the *Arabidopsis* GOX3 overexpression plants which were shown to be more tolerant to L-lactate toxicity [22]. As a semi-aquatic plant, rice produces low lactate as compared with wheat, potato and *Arabidopsis* [35–38], which might explain why GLO3 is not associated with lactate toxicity in rice as reported for *Arabidopsis* [22].

While the primary metabolic role of GLO is well known, the physiological function is still not well understood. Rojas et al. (2012) suggested that each GLO isozyme played different roles in the non-host disease resistance in *Arabidopsis* [16]. A more recent study demonstrated that the *Arabidopsis* GOX1 and GOX2 played distinct roles in the oxidative stress-related cell death [23]. GLO has also been reported to be involved in some other biological processes such as protein repair responses and salicylic acid signaling pathway [39, 40]. More interestingly, recent studies found that there was cross-talk between photorespiratory H_2_O_2_ and auxin [41, 42]. We found that GLO activity was closely related to H_2_O_2_ levels in rice leaves (Additional file 5), and, as such, the dwarfism phenotype of the GLO down-regulated rice lines could be a morphological aberration related to auxin signaling [43, 44]. Nevertheless, our previous work demonstrated that suppression of GLO may cause accumulation of glyoxylate that inhibits photosynthesis [5], so it is also possible that the dwarfism phenotype results from inhibited photosynthesis. Therefore, each GLO isozyme may exhibit different physiological functions in these various biological processes in rice, but more direct experimental evidence is needed to elucidate the potential mechanisms.

## Conclusions

Our findings suggested that rice GLO isozymes have distinct enzymatic characteristics and different physiological functions. GLO1, GLO4 and GLO1 + 4 are the photorespiration GLO isozymes, moreover, GLO1 is the major contributor to GLO activity and the related photorespiratory H_2_O_2_ production. In addition, there may be interplay between the photorespiration glycolate-H_2_O_2_ metabolism and plant development. However, the certain functions of GLO3 and GLO5 remain to be fully elucidated.

## Methods

### Plant materials and growth conditions

The seeds of rice (*Oryza sativa*) cv. Zhonghua 11 (japonica cultivar-group) provided by the state key laboratory for conservation and utilization of subtropical agro-bioresources were used for the construction of transgenic lines. Rice seeds were germinated in the dark for 4-6 days at 25 °C, and then the seedlings were grown in Kimura B complete nutrient solution [45] in plant growth chambers with 14 h light (30 °C) /10 h dark (25 °C), 800 μmol m^−2^ s^−1^ average light intensity, and 60-70% relative humidity. After reaching the 4-leaf stage, seedlings were transplanted, either being continuously grown in Kimura B complete nutrient solution in the plant growth chambers, or grown in soil under natural condition. The seedlings grown in Kimura B complete nutrient solution in plant growth chambers were exposed to various stress treatments (PEG 6000, 10%; NaCl, 100 mM; ABA, 10 μM; H_2_O_2_, 5 mM; AlCl_3_, 2 mM). The seedlings grown in soil under natural condition were used for GLO isozyme zymogram and growth phenotype analyses.

### Plasmid construction

Total RNA was extracted from rice leaves using RNAprep Pure Kit (TIANGEN, China). The quality and quantity of the purified RNA were assessed with a NanoDrop-1000 (NanoDrop, USA). First-strand cDNA was synthesized using ReverTra Ace (Toyobo, Japan). Primers were designed to cover the complete open reading frame of each *GLO* gene (Additional file 8). To construct the vectors for protein expression in yeast, a 6 × His-tag was fused to the N-terminus of *GLO1*, *GLO3* and *GLO4* [23], and then these modified sequences (*NHisGLO1*, *NHisGLO3* and *NHisGLO4*) were cloned into pYES3 and pYES2 vectors. To generate *GLO-*overexpression transgenic lines, each *GLO* sequence was cloned into pYLox.5 vector. To generate *GLO-*silencing transgenic lines, primers were designed to amplify the interfering fragment to guarantee the specificity of the silencing (Additional file 8), each specific fragment was then cloned into the RNAi vector pYLRNAi.5. To generate CRISPR-Cas9 knockout lines, specific targeting sequences were synthesized and cloned into pYLCRISPR/Cas9P_ubi_ vector (Additional file 4) [46]. (pYLox.5, pYLRNAi.5 and pYLCRISPR/Cas9 vectors were kindly provided by Dr. Yao-Guang Liu, College of Life Sciences, South China Agricultural University).

### Protein expression in *Saccharomyces cerevisiae*

The constructed GLO expression vectors were transformed into *Saccharomyces cerevisiae* INVSc1 (*his3Δ1/his3Δ1 leu2/leu2 trp1-289/trp1-289 ura3-52/ura3-52*, Invitrogen) using the lithium acetate/carrier DNA method [47]. After that, yeast cells transformed with GLO constructs were maintained in the SC selective plates (SC-Dropout Medium without Trp/Ura) with 2% glucose, at 30 °C for 3-4 days to isolate the positive clones. The proteins were inductively expressed in *S.cerevisiae* as previously described [24, 48] with some modifications. Briefly, positive clones were transferred to 10 mL SC selective medium containing 2% glucose, and incubated at 30 °C for 16 h with shaking at 250 rpm. Appropriate volumes of the culture were transferred to 50 mL SC inductive culture medium (SC selective medium containing 2% galactose) to obtain an OD600 of 0.4, and then shaken at 30 °C with 250 rpm for 24 h. Cells were harvested by centrifuging the culture at 5000 rpm for 5 min at 4 °C, the cell pellets were collected and stored at −75 °C until ready to use.

### Western blot analysis

Proteins were extracted from yeast cells using acid-washed glass beads as described previously [48]. Supernatant of the yeast cells extract was collected and used for SDS polyacrylamide gel (SDS-PAGE) electrophoresis. Protein samples separated on 10% SDS-PAGE were electroblotted onto a nitrocellulose membrane using wet transfer [49, 50]. GLO was detected using a GLO-antibody. The anti-GLO primary antibody was generated in rabbits by immunization with GLO1 proteins, and this antibody was able to recognize all the rice GLO polypeptides due to the high similarity between these polypeptides.

### Purification of active GLO from yeast cells

Supernatant of the yeast cell lysate was filtered through a 0.45 μm membrane for subsequent purification. The Ni-IDA resin (Bio-Rad) was packed in a Bio-Scale MT5 column (10 × 64 mm) for bed volumes up to 2 mL, equilibrate the column with 5 column volumes (CV) of wash buffer (50 mM PBS, pH 8.0, 10 mM imidazole, 300 mM NaCl). The filtered supernatant were prepared by mixing them with an equal volume of binding buffer (100 mM PBS, pH 8.0, 20 mM imidazole, 600 mM NaCl), and then loading them onto the Ni-IDA resin column. The column was washed with 10 CV of wash buffer at a flow rate of 1.0 mL min^−1^. The bound proteins were eluted with 5 CV of 50 mM PBS (pH 8.0) containing 150 mM imidazole and 300 mM NaCl. The eluted fractions were desalted by ultrafiltration and checked by 10% SDS-PAGE. All the purified enzymes were stored in 50 mM PBS (pH 8.0) containing 0.1 mM FMN and 10% glycerol at −75 °C for subsequent assay.

### GLO isozyme zymogram analysis

To identify GLO isozymes a Caps-ammonium Clear Native-PAGE system (CN-PAGE) with a running pH of 10.2 was used [51]. The separation gel concentration is 6%, the electrophoresis was performed at 100 V for 9 h at 4 °C. Activity staining was carried out by incubating the gel at 30 °C for 20 min in a staining solution which contained 0.016% (*W*/*V*) NBT, 0.003% (*W*/*V*) PMS,0.1 mM FMN,10 mM glycolate (pH 6-8),and 50 mM PBS (pH 8.0).

### GLO activity assay

GLO catalytic activity was measured in an enzyme-coupled assay according to Hall et al. (1985) unless specific variations were needed in different kinetic and characterization investigations [52]. The typical reaction mixture containing 50 mM PBS (pH 7.8), 5 units of horseradish peroxidase, 1 mM 4-amino-antipyrine, 2 mM phenol, 0.1 mM FMN and 10 mM substrate. The reaction was started by adding the enzyme, distilled water was substituted for substrate as the blank. Formation of H_2_O_2_ produced in the reaction which reflects the catalytic activity of GLO was monitored spectrophotometrically at 520 nm under 30 °C.

The effects of varying pH were assayed with two buffers: pH of 50 mM PBS from 6 to 8, and 50 mM Tris-HCl from 8 to 9. As for the effects of temperature, the reaction mixtures were pre-incubated at various temperatures (22-60 °C) for 5 min, and then the GLO activities were measured at the same temperature.

For kinetic parameter determination, oxidations of the substrate were performed over a range of concentration (0.1-0.8 mM for glycolate, 1.0-8.0 mM for glyoxylate, 0.5-6.0 mM for L-lactate, 0.5-6.0 mM for glycerate), the *K*_*m*_ was calculated from double-reciprocal plots according to the method of Lineweaver and Burk, moreover, slopes of the reciprocal plots were then plotted against the concentration of oxalate (range 1.0-8.0 mM), to evaluate *K*_*i*_ data [53].

### Generation of GLO transgenic line

The constructed vectors were introduced into rice by *Agobacterium*-mediated infection (strain EHA105) [54]. The seeds from the positive T0 lines were germinated in complete Kimura B nutrient solution and then transplanted to soil. The T2 or T3 plants were used for the determination of GLO activity and the Real-time quantitative PCR (qRT-PCR) assay. Besides, the T3 plants of GLO3-transgenic lines were used for lactate toxicity test (L-lactate, 2.0 mM).

### Real-time quantitative PCR

The specific primer pairs were designed for the qRT-PCR of each *GLO* gene (Additional file 8). Total RNA was purified from rice using TRIzol® reagent (Life Technologies, Carlsbad, USA), and further treated with DNase I (RNase free, Toyobo, Osaka, Japan). The quality of the isolated RNA was assessed with a NanoDrop-1000 (Thermo Fisher Scientific, Bremen, Germany). One microgram of RNA was used as a template for first-strand cDNA synthesis using ReverTra Ace (Toyobo, Osaka, Japan). The qRT-PCR reaction mixture consisted of 0.2 μM (each) primer, 10 μL of 2 × SYBR Green PCR Master Mix (Toyobo, Osaka, Japan), and 2 μL of appropriate diluted cDNA. The analysis was conducted using a DNA Engine Opticon 2 Real-Time PCR Detection system and Opticon Monitor software (Bio-Rad, Hercules, CA). The data were normalized to the amplification of the *OsActin1* gene (Os03g0718100).

### 3, 3′-diaminobenzidine staining

The leaf H_2_O_2_ abundance was estimated by the 3, 3′-diaminobenzidine (DAB) uptake method [55]. The youngest fully expanded leaves of the five-leaf stage rice were detached (10 cm), and the cut end was dipped into 4 mL of DAB solution (1 mg mL^−1^, pH 3.8) for 2 h in light at 30 °C. The experiment was terminated by boiling the leaves in ethanol for 30 min.

### Quantification of proteins

The protein content was determined according to Bradford (1976) with bovine serum albumin as a standard [56], and the experiments were repeated three times with at least three replicates for each sample.

## Additional files

Additional file 1: Similarities of rice *GLO* gene members at the level of mRNA and protein. (DOCX 16 kb)

Additional file 2: (a) Effect of varying pH on activities of GLO isozymes. Each buffer (50 mM) was made of respective PBS (pH 6.0-8.0) and Tris-HCl (pH 8.0-9.0). (The highest activity of each GLO isozyme at pH 7.8 was set as 1). (b) Effect of temperature on activities of GLO isozymes. Enzymes in 50 mM PBS buffer (pH 7.8) were pre-incubated at various temperatures (22-60 °C) for 5 min, and then activities were measured at the same temperature (For GLO1 and GLO1 + 4, the highest activity at 45 °C was set as 1; For GLO3, the highest activity at 47 °C was set as 1; For GLO4, the highest activity at 42 °C was set as 1). Values are means ± SD (*n* = 3). (TIFF 7340 kb)

Additional file 3: The *V*_*max*_ of purified GLO isozymes with various substrates. (DOCX 18 kb)

Additional file 4: (a) Relative mRNA levels were graphed based on the *GLO1* mRNA level in WT leaves as 1. (b) Relative mRNA levels were graphed based on the *GLO4* mRNA level in WT leaves as 1. (c) Mutation of GLO1 and GLO4 knockout lines generated by pYLCRISPR/Cas9P_ubi_ system. (d) Relative mRNA levels were graphed based on the *GLO3* mRNA level in WT leaves as 1. (e) Relative mRNA levels were graphed based on the *GLO5* mRNA level in WT leaves as 1. Values are means ± SD (*n* = 3). (TIFF 5131 kb)

Additional file 5: H_2_O_2_-3, 3′-diaminobenzidine (DAB) staining in rice leaves. Cas9-GLO1 and Cas9-GLO4 represent the GLO1 and GLO4 knockout plants, respectively. The result is representative of three independent experiments. (TIFF 10179 kb)

Additional file 6: L-lactate toxicity test in GLO3 transgenic plants. Different transgenic rice lines (4-leaf stage) were grown in Kimura B complete nutrient solution containing 2.0 mM L-lactate for one week. OX-GLO3 and Ri-GLO3 represent the GLO3 overexpression transgenic plants and the specific GLO3 RNA-silencing transgenic plants, respectively. The results are representative of three independent experiments. (TIFF 7069 kb)

Additional file 7: GLO activity in root of GLO3 over expression plants. (DOCX 17 kb)

Additional file 8: The primers used for the plasmid construction and real-time quantitative PCR. (DOCX 18 kb)

## Abbreviations

2-PG: Phosphoglycolate
CN-PAGE: Clear-native polyacrylamide gel electrophoresis
DAB: 3, 3′-diaminobenzidine
FMN: Flavin mononucleotide
GLO: Glycolate oxidase
PGP: 2-PG phosphatase
qRT-PCR: Real-time quantitative PCR
