# Supplementary material for: Catalytic and functional aspects of different isozymes of glycolate oxidase in rice
Source: BMC Plant Biol. 2017 Aug 8;17:135. doi: 10.1186/s12870-017-1084-5 (PMC5549332; doi:10.1186/s12870-017-1084-5)
Supplement: Supplementary file 1 — Similarities of rice GLO gene members at the level of mRNA and protein. (DOCX 16 kb) [file 12870_2017_1084_MOESM1_ESM.docx]

Additional file 1: Similarities of rice *GLO* gene members at the level of mRNA and protein

| Protein  mRNA | GLO1 | GLO3 | GLO4 | GLO5 |
| --- | --- | --- | --- | --- |
| *GLO1* |  | 83.3% | 89.7% | 58.9% |
| *GLO3* | 75.6% |  | 85.1% | 57.9% |
| *GLO4* | 86.8% | 74.9% |  | 58.1% |
| *GLO5* | 62.6% | 58.6% | 60.6% |  |
