# Supplementary figures and images for: Catalytic and functional aspects of different isozymes of glycolate oxidase in rice
Source: BMC Plant Biol. 2017 Aug 8;17:135. doi: 10.1186/s12870-017-1084-5 (PMC5549332; doi:10.1186/s12870-017-1084-5)

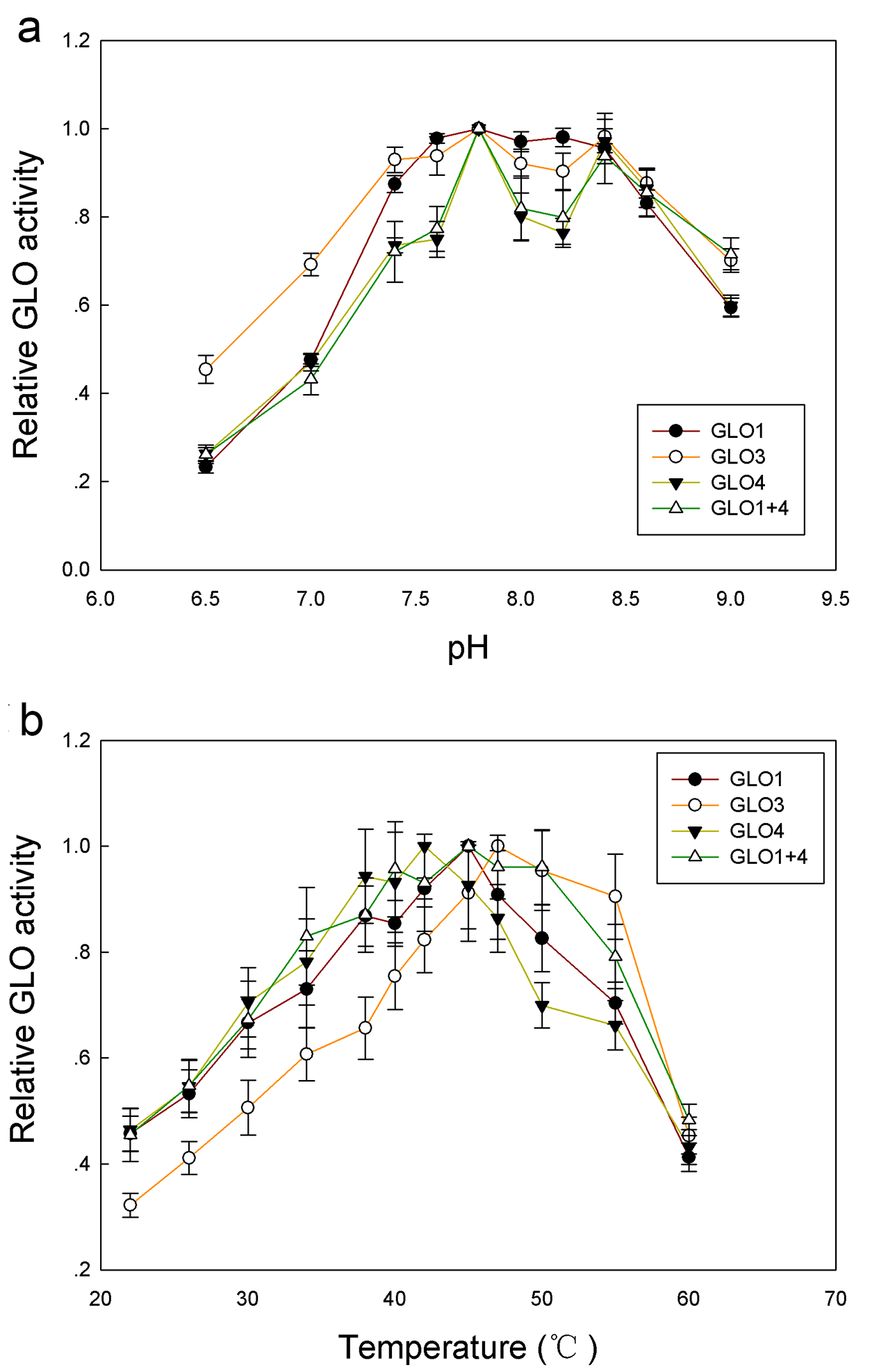

Supplement: Supplementary file 2 — (a) Effect of varying pH on activities of GLO isozymes. Each buffer (50 mM) was made of respective PBS (pH 6.0-8.0) and Tris-HCl (pH 8.0-9.0). (The highest activity of each GLO isozyme at pH 7.8 was set as 1). (b) Effect of temperature on activities of GLO isozymes. Enzymes in 50 mM PBS buffer (pH 7.8) were pre-incubated at various temperatures (22-60 °C) for 5 min, and then activities were measured at the same temperature (For GLO1 and GLO1 + 4, the highest activity at 45 °C was set as 1; For GLO3, the highest activity at 47 °C was set as 1; For GLO4, the highest activity at 42 °C was set as 1). Values are means ± SD (n = 3). (TIFF 7340 kb) [file 12870_2017_1084_MOESM2_ESM.tif]

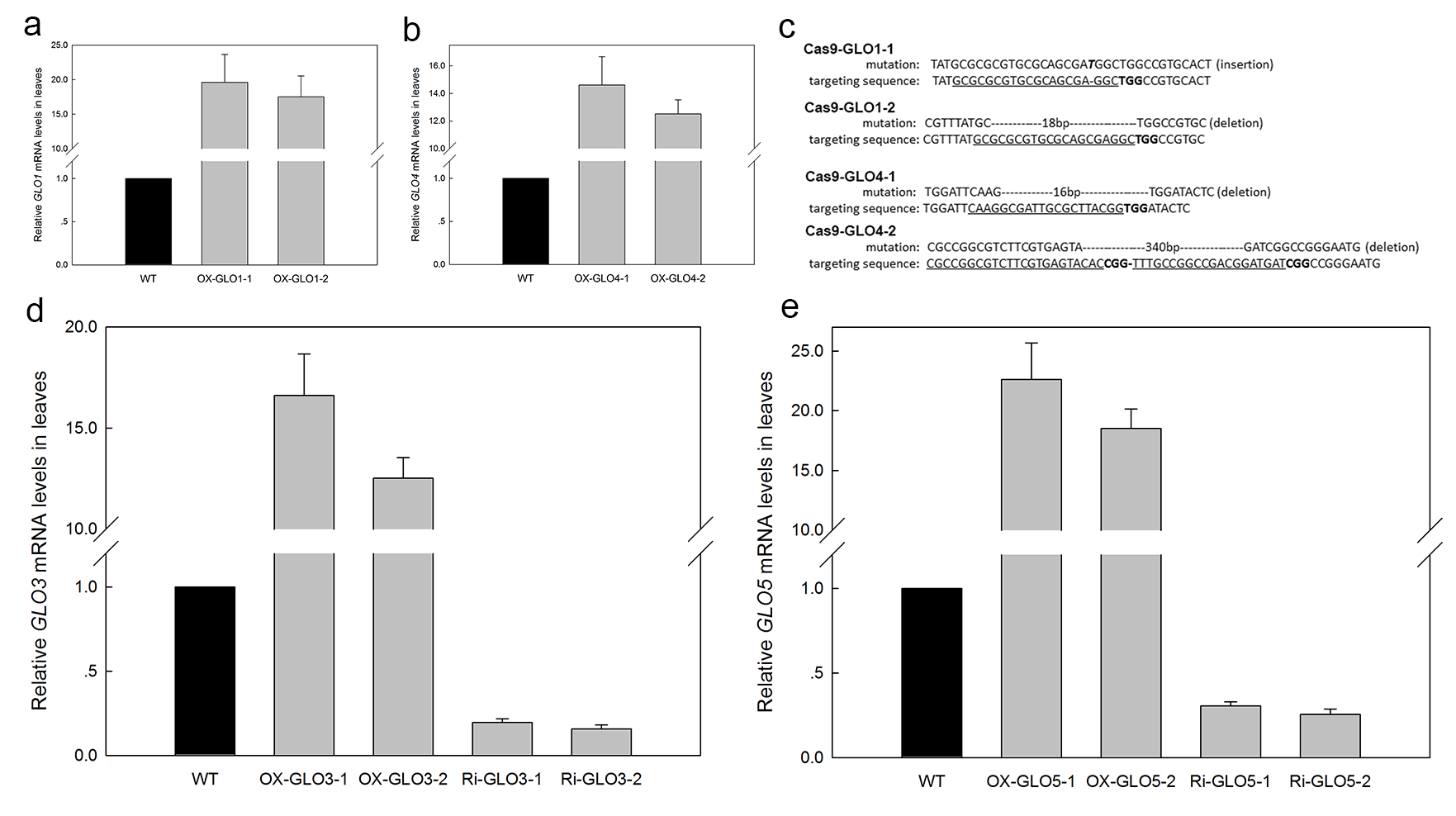

Supplement: Supplementary file 4 — (a) Relative mRNA levels were graphed based on the GLO1 mRNA level in WT leaves as 1. (b) Relative mRNA levels were graphed based on the GLO4 mRNA level in WT leaves as 1. (c) Mutation of GLO1 and GLO4 knockout lines generated by pYLCRISPR/Cas9Pubi system. (d) Relative mRNA levels were graphed based on the GLO3 mRNA level in WT leaves as 1. (e) Relative mRNA levels were graphed based on the GLO5 mRNA level in WT leaves as 1. Values are means ± SD (n = 3). (TIFF 5131 kb) [file 12870_2017_1084_MOESM4_ESM.tif]

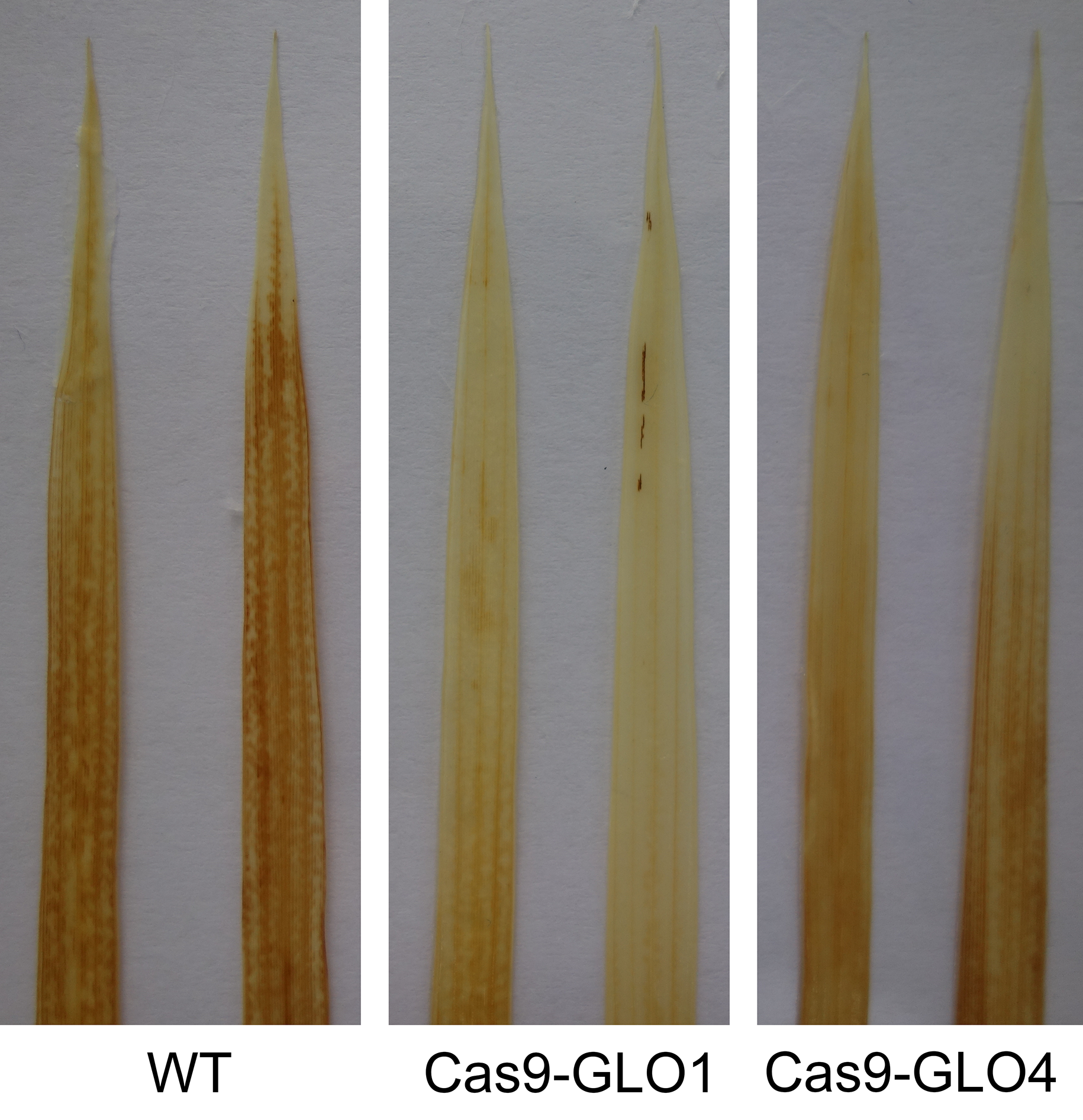

Supplement: Supplementary file 5 — H2O2-3, 3′-diaminobenzidine (DAB) staining in rice leaves. Cas9-GLO1 and Cas9-GLO4 represent the GLO1 and GLO4 knockout plants, respectively. The result is representative of three independent experiments. (TIFF 10179 kb) [file 12870_2017_1084_MOESM5_ESM.tif]

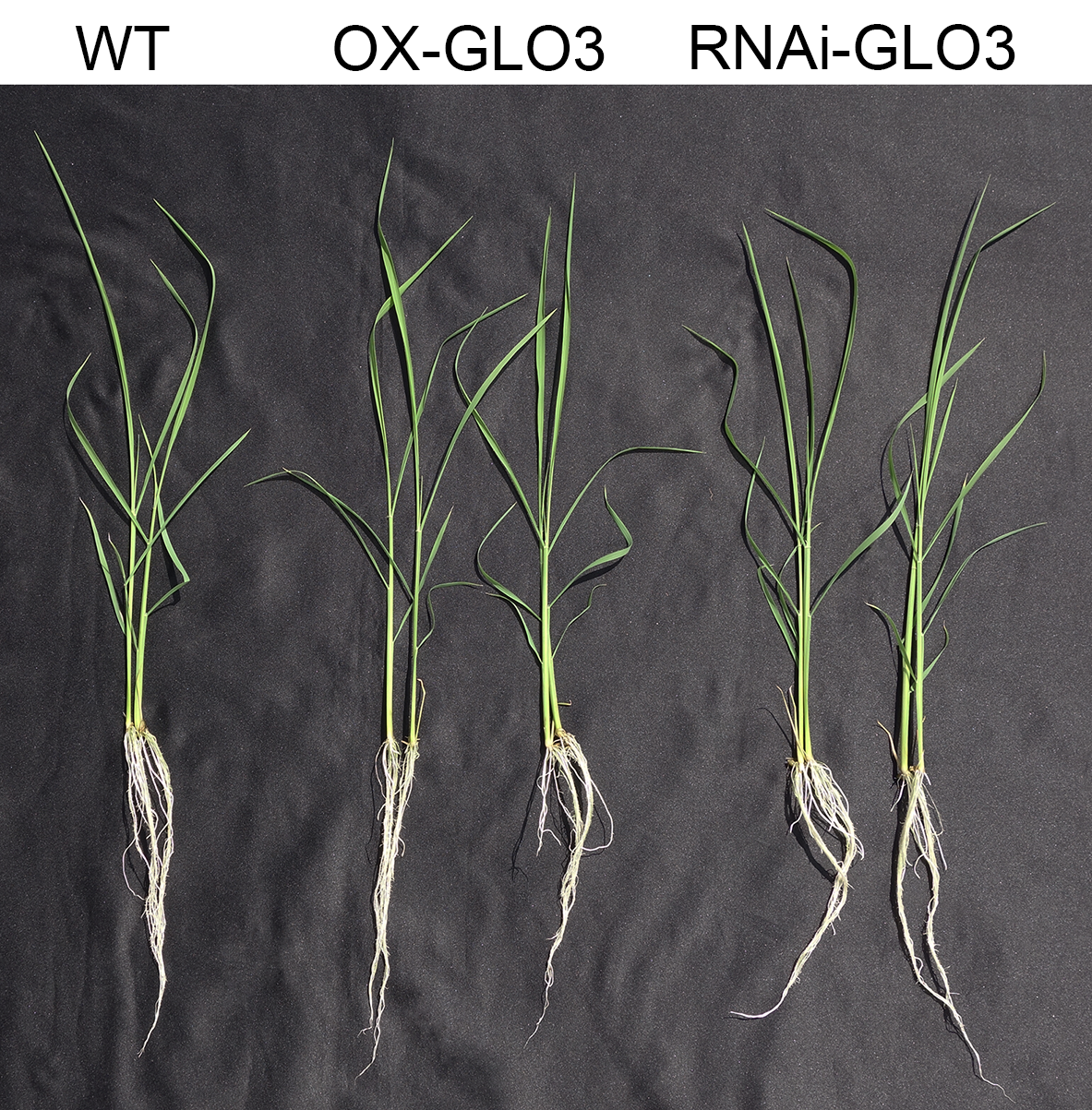

Supplement: Supplementary file 6 — L-lactate toxicity test in GLO3 transgenic plants. Different transgenic rice lines (4-leaf stage) were grown in Kimura B complete nutrient solution containing 2.0 mM L-lactate for one week. OX-GLO3 and Ri-GLO3 represent the GLO3 overexpression transgenic plants and the specific GLO3 RNA-silencing transgenic plants, respectively. The results are representative of three independent experiments. (TIFF 7069 kb) [file 12870_2017_1084_MOESM6_ESM.tif]
