# Supplementary material for: Catalytic and functional aspects of different isozymes of glycolate oxidase in rice
Source: BMC Plant Biol. 2017 Aug 8;17:135. doi: 10.1186/s12870-017-1084-5 (PMC5549332; doi:10.1186/s12870-017-1084-5)
Supplement: Supplementary file 3 — The V max of purified GLO isozymes with various substrates. (DOCX 18 kb) [file 12870_2017_1084_MOESM3_ESM.docx]

Additional file 3: The *V_max_* of purified GLO isozymes with various substrates

| GLO  isoenzymes | *V_max(glycolate)_*  (μmol glycolate min^-1^ mg^-1^)  protein | *V_max(glyoxylate)_*  (μmol glyoxylate min^-1^ mg^-1^)  protein | *V_max(L-lacatate)_*  (μmol glyoxylate min^-1^ mg^-1^)  protein | *V_max(glycerate)_*  (μmol glyoxylate min^-1^ mg^-1^)  protein |
| --- | --- | --- | --- | --- |
| GLO1 | 63.583±5.531 | 9.240±1.343 | 23.229±3.349 | 42.943±4.513 |
| GLO3 | 32.197±3.272 | 4.486±0.883 | 52.871±7.449 | 5.107±0.669 |
| GLO4 | 45.959±5.107 | 6.235±1.045 | 21.585±1.874 | 29.716±2.359 |
| GLO1+4 | 80.244±7.046 | 7.892±1.158 | 38.235±2.016 | 51.718±4.912 |

Values are means ±SD of three replicates.
