# Supplementary material for: Catalytic and functional aspects of different isozymes of glycolate oxidase in rice
Source: BMC Plant Biol. 2017 Aug 8;17:135. doi: 10.1186/s12870-017-1084-5 (PMC5549332; doi:10.1186/s12870-017-1084-5)
Supplement: Supplementary file 7 — GLO activity in root of GLO3 over expression plants. (DOCX 17 kb) [file 12870_2017_1084_MOESM7_ESM.docx]

Additional file 7: GLO activity in root of GLO3 over expression plants

| GLO activity  Rice line | Glycolate-oxidation  μmolH_2_O_2_ min^-1^ mg^-1^ protein | L-lactate-oxidation  μmolH_2_O_2_ min^-1^ mg^-1^ protein |
| --- | --- | --- |
| WT | ND | ND |
| OX-GLO3-1 | 0.051±0.012 | 0.135±0.035 |
| OX-GLO3-2 | 0.045±0.019 | 0.116±0.021 |

Values are means ±SD of three replicates. ND indicates “not detected”.
