# Supplementary material for: Catalytic and functional aspects of different isozymes of glycolate oxidase in rice
Source: BMC Plant Biol. 2017 Aug 8;17:135. doi: 10.1186/s12870-017-1084-5 (PMC5549332; doi:10.1186/s12870-017-1084-5)
Supplement: Supplementary file 8 — The primers used for the plasmid construction and real-time quantitative PCR. (DOCX 18 kb) [file 12870_2017_1084_MOESM8_ESM.docx]

Additional file 8: The primers used for the plasmid construction and real-time quantitative PCR

| Gene | Primer sequences |
| --- | --- |
| *GLO1*-pYES3 | GLO1-F1: 5-ATGCATCATCACCATCACCATatgggggagatc-3  GLO1-R1: 5-AGTCGAATTCtcacaacctggggaaggG-3  GLO-F2: 5-GCGCGGTACCATGCATCATCACCATatg-3 |
| *GLO3*-pYES3 | GLO3-F1: 5-ATGCATCATCACCATCACCATatggagctaatc-3  GLO3-R1: 5-TATACTCGAGctacagcctggagcggcg-3  GLO-F2: 5-GCGCGGTACCATGCATCATCACCATatg-3 |
| *GLO4*-pYES3 | GLO4-F1: 5-ATGCATCATCACCATCACCATatgggggagatc-3  GLO4-R1: 5-GCACGAATTCttacaagcgagacggcatg-3  GLO-F2: 5-GCGCGGTACCATGCATCATCACCATatg-3 |
| *GLO5*-pYES3 | GLO5-F1: 5-ATGCATCATCACCATCACCATatggaggacaat-3  GLO5-R1: 5-TGTACTCGAGtcagagcagggacctgat-3  GLO-F2: 5-GCGCGGTACCATGCATCATCACCATatg-3 |
| *GLO3*-RNAi | GLO3-RNAi-F: 5-AGGAAGCTTAGGCTAAGCTCTTCAGCGGTTG-3  GLO3-RNAi-R:5-CCCGGATCCCAAACTACTCTGAATCACCAAAT-3 |
| *GLO5*-RNAi | GLO-5-RNAi -F: 5-TTAgAgCTCCgCCATAATggTTCTTTCC-3  GLO-5-RNAi-R: 5-gTTAAgCTTAgCggTgACgATgCCCTT-3 |
| *GLO1*-qRT | GLO-1-qRT-F: 5-TCGTTCTGCCACCATACTTG-3  GLO-1-qRT-R: 5-GCCACTTCACATCCTTCCAG-3 |
| *GLO3*-qRT | GLO-3-qRT-F: 5-TGATGCCGGGTAATAAGGAG-3  GLO-3-qRT-R: 5-CAACGACAGCAACAGTGACA-3 |
| *GLO4*-qRT | GLO-4-qRT-F: 5-CACACCCTCCATTTCTTGCT-3  GLO-4-qRT-R: 5-GCCGAATCACCTACACCACT-3 |
| *GLO5*-qRT | GLO-5-qRT-F: 5-CGTTGCCAAAGATGGCTTAT-3  GLO-5-qRT-R: 5-CAAGAACGCGAGGTCGTAAT-3 |
| *OsActin1*-qRT | Actin1-qRT-F: 5-CTTCATAGGAATGGAAGCTGCG-3  Actin1-qRT-R: 5-CACCTTGATCTTCATGCTGCTA-3 |
